# Investigating Caesarean Section Practice among Teenage Romanian Mothers Using Modified Robson Ten Group Classification System

**DOI:** 10.3390/ijerph182010727

**Published:** 2021-10-13

**Authors:** Alexandra Matei, Mihai Cornel Dimitriu, George Alexandru Roșu, Cristian George Furău, Crîngu Antoniu Ionescu

**Affiliations:** 1 Department of Obstetrics and Gynecology, “Carol Davila” University of Medicine and Pharmacy Doctoral School, 020021 Bucharest, Romania; antoniuginec@yahoo.com; 2 Department of Obstetrics and Gynecology, “Sf. Pantelimon” Emergency Clinical Hospital, 021659 Bucharest, Romania; mihai.dimitriu@umfcd.ro (M.C.D.); george.rosu@ymail.com (G.A.R.); 3 Department of Obstetrics and Gynecology, Faculty of Medicine, “Vasile Goldiș” Western University of Arad, 310025 Arad, Romania; cristianfurau@gmail.com

**Keywords:** Robson classification, caesarean section, caesarean indication, adolescent birth, pregnant women

## Abstract

The Robson ten-group classification system is a recognized effective method of assessing caesarean rate. It is based on dividing patients into ten mutually exclusive groups, focusing on six maternal and newborn variables (parity, gestational age, plurality, foetal presentation, previous caesarean, and mode of labour onset). The aim of our analysis was twofold: first, to present the implementation of Robson classification in a pregnant teenage population; and second, to identify the indications for CS in the adolescent population. This study was designed as a one-year prospective analysis and considered all women younger than 20 years of age who delivered in a tertiary care hospital. Before discharge, women who had caesarean delivery responded to a questionnaire regarding their education, prenatal surveillance, and obstetrical history. Caesarean sections accounted for 47.01% of all births. A proportion of 24.57% of the participants had at least one previous caesarean section. Group 10 (all women with a single cephalic preterm pregnancy) was second most often identified among women in middle adolescence (14.03%); 32.20% of the participants in late adolescence were in group 5 (multiparas with a scarred uterus, single cephalic term pregnancy). Differences between the two age groups were not statistically different (*p* = 0.96). Abnormal cardiotocographic findings (38.23%), the arrest of descent (19.11%) and arrest of dilation (19.11%), were the most frequent indications for caesareans in Robson group 1. Neonates from mothers in Robson groups 8 (women with a multiple pregnancy) and 7 (multiparas single breech pregnancy) had the most unfavourable outcomes regarding gestational age at delivery and admission to the intensive care unit. We concluded that future focus on obstetrical management is mandatory in Robson groups 7 and 8. Adolescents in Robson group 1 (nulliparas, single cephalic term pregnancy, spontaneous labour) are the primary beneficiaries of strategies to reduce caesarean sections rates.

## 1. Introduction

In 2018, the caesarean section (CS) was the second most common surgical procedure conducted in the European Union hospitals after cataract surgery, being performed at least 1.4 million times [1,2]. The year before, Cyprus and Romania were the two countries reporting the highest rates of CS *54.8% and 44.1%, respectively), whereas in Finland only 16.5% of all live births came through CS [2]. These prominent differences have been suggested to be related to a variety of factors, ranging from extreme maternal age and their associated obstetrical comorbidities, to the clinician’s experience, fear of litigation or the financial management of maternity care; the economic, social and cultural environment; and the women’s preference and education are also determinant factors for CS practice [3,4,5]. However, overall CS rates conflate multiple groups associating differing levels of risk. 

In Romania, particularly, adolescent pregnancy and birth are a matter of public health issue. Although over the past five years the number of births among 15 to 19 years old teenagers has been decreasing, there is concern regarding childbearing in mothers aged 10 to 14 years old, where official reports identified a 3.31% increase in 2019 compared to 2018 [6]. Assessment of maternal perinatal morbidity and mortality in these groups of pregnant young women is crucial, especially in our country where they account for 9.35% of total live births [6]. In this context, it is also relevant to identify the contribution of this vulnerable population to the overall national CS rates. Previous Romanian studies have published engaging results related to adolescent birth. While specific customs, traditions, poverty and young women’s lack of education were identified as linked to teenage pregnancy, other authors concluded that mothers of teenage pregnant women were proven to have good knowledge about reproductive education [4,7].

Although obstetric practice in any healthcare facility is affected by factors such as availability of resources and clinical management protocols [8], the rise in CS rates has become a global phenomenon of multifactorial aetiology, associating short- and long-term complications, extending beyond current delivery, and affecting future pregnancies as well. 

Performing an analysis on indications for CS is ambiguous, as some deliveries can associate two or more indications. The Robson ten-group classification system (TGCS) has been proposed by the World Health Organization (WHO) as the global standard for assessing, monitoring, and comparing CS rates [8], which is clinically easy to apply and understand. It has been designed as a mutually exclusive and totally inclusive tool [9], thereby offering insight on specific epidemiological factors, obstetrical events and outcomes involved in all deliveries (and particularly in CS). TGCS can provide a homogeneous framework for analysing CS rates and their implication in obstetrical practice. It has been proven that CS rates > 10% do not associate any reductions in maternal and neonatal mortality [8]. Therefore, it is essential to investigate the real cause of this rise and whether this affects younger mothers as well. There are five obstetric concepts involved in the TGCS classification: parity, gestational age, the category of the pregnancy (single or multiple), foetal presentation, the previous obstetric record of the woman, and the mode of labour onset [9]. These are clinically relevant to obstetricians and can provide comprehension within healthcare facilities as well as between them. 

Considering our country’s debut of transitioning towards lower maternal age at delivery and the associated financial burden related to increased CS use and corresponding morbidity, the authors consider that specific attention is required for analysing local obstetrical practices. 

Adolescent population and underage mothers, particularly, raise significant medical and legal concerns related to their decision-making autonomy. Most young women come from dysfunctional families, and it is not uncommon for obstetricians to experience situations when no parent or legal guardian can be consulted for life-saving medical decisions involving young mothers. Adding potential fear of litigation, a determining factor involved in practising defensive medicine, the necessity for assessing the boundary between medically necessary CS and disruptive practice is justified. 

This study aims to address the issue of CS among pregnant teenagers by assessment of data from one obstetrical unit. The primary outcome of our analysis is to describe the obstetric framework using the TGCS implemented in this distinctive group of patients. The secondary outcome is based on identifying the indications for CS in the adolescent population. The novelty of this research resides in the assessment of TGCS in adolescent mothers. 

This research was possible with hospital ethics committee approval (2134/21 January 2020). All procedures performed in this study were following the ethical standards of the institutional ethics committee and with the 1964 Helsinki 60 declaration and its later amendments or comparable ethical standards.

## 2. Materials and Methods

We aimed to perform a Romanian single-centre analysis of CS rates based on TGCS reported in adolescent mothers. The secondary distinctive outcome was to describe the indications for CS use, to gain a better insight into teenage obstetrical practice.

This study was developed in the “Sf. Pantelimon” Emergency Hospital in Bucharest, Romania, a tertiary medical care unit registering approximately 1600 births per year. The analysis has been performed prospectively, for one-year, from 1 March 2020 to 1 March 2021. Maternal age was defined as age at birth. We have considered adolescents to be all women 10 to 20 years of age. Adolescents who delivered in our department during the study period and signed the informed consent to participate in our research have been included in the analysis. Women aged 20 years old and above, those younger than 10 years old, and those who refused to participate in the study were excluded from the research protocol. Young women under 16 years of age were automatically monitored during hospital admission by investigators from the social services and child protection services division.

The individual group of young women who delivered through CS was assigned as a “study group” and was subdivided into three different age groups: 10 to 14 years old, known as early adolescence; 15 to 17 years old, known as middle adolescence; and 18 to 19 years old, also known as late adolescence (according to WHO) [10]. For these women, the corresponding newborn variables were collected, namely newborn birth weight, Apgar score and admission to a neonatal intensive care unit (NICU).

The following sources were used to collect the parameters included in the database used for this research:

- Questionnaires for maternal residence (rural or urban), level of education expressed in completed years of schooling, number of prenatal visits, parity, and number of previous CS;
- Birth registries, maternal variables, gestational age expressed in weeks, type of pregnancy (single or multiple), foetal presentation (cephalic or breech), indication for CS, ad newborn variables, newborn birthweight expressed in grams, and 5-min Apgar score;
- Electronic admission charts for maternal hospital length of stay and newborn NICU length of stay (both variables expressed in days).

Rural settlements were the smallest administrative-territorial units, characterized by a reduced density of population, where agricultural occupations are performed. Women who deliver through CS spend in the hospital four to five days on average. We have considered a length of stay ≥ five days to be an indirect but suggestive indicator of maternal and/or neonatal outcomes.

Robson scores were introduced for all young mothers irrespective of their mode of delivery. The specific scores were obtained from birth registries.

Neonates having complications at birth such as respiratory depression were included in the statistical analysis using a 5-min Apgar score variable less than 7. 

The objective of this research is to highlight the relevance of using TGCS in obstetrical practice. Additionally, we have assessed standard guideline CS indications and neonatal outcomes. Currently, the Robson classification is not implemented nationally. Although in our setting perinatal audit is constantly progressing, the process of raw data transformation into medical protocols is laborious and lingering. TGCS was the resolution for our practice, acknowledging that it was initially designed to analyse labour and birth events in the context of specific types of management that each unit may have. 

To ensure the quality of perinatal data collection, TGCS was implemented in our department using the national guideline recommendation on the system classification; in the context of significant CS rates, groups 5 to 10 were further subdivided [11]. A complementary reason for this was the need for overlap-bias reduction concerning the mode of labour onset. We have considered the template as modified TGCS (mTGCS0 and was used as follows:

- Robson group 1:
  Nulliparous women, single cephalic, ≥37 weeks, in spontaneous labour
- Robson group 2:
  Nulliparous women, single cephalic, ≥37 weeks, with:
  - Induced labour
  - CS before labour
- Robson group 3:
  Multiparous women excluding previous CS, single cephalic, ≥37 weeks, in spontaneous labour
- Robson group 4:
  Multiparous, singleton, cephalic, without previous CS, with:
  - Induced labour
  - CS before labour
- Robson group 5:
  Multiparous women, previous CS, single cephalic, ≥37 weeks, with:
  1. One previous CS
  2. Two or more previous CS
    - Spontaneous labour
    - Induced labour
    - CS before labour
- Robson group 6:
  All nullipara breeches with:
  - Spontaneous labour
  - Induced labour
  - CS before labour
- Robson group 7:
  All multipara breeches, including previous CS, with:
  - Spontaneous labour
  - Induced labour
  - CS before labour
- Robson group 8:
  All multiple pregnancies with:
  - Spontaneous labour
  - Induced labour
  - CS before labour
- Robson group 9:
  Transverse and oblique lies, including previous CS with:
  - Spontaneous labour
  - Induced labour
  - CS before labour
- Robson group 10:
  All single cephalic, ≤36 weeks, including previous CS, with:
  - Spontaneous labour
  - Induced labour
  - CS before labour

We have included in the study data from all young women who met the inclusion criteria in order to assess properly each group from the mTGCS and to report the corresponding relative sizes. Particularly, we have performed an in-depth analysis of teenagers who delivered through CS by applying a questionnaire focusing on their residence, education, prenatal medical surveillance and scheduled appointments, obstetrical history, parity, and previous CS. The questionnaire was implemented by an assigned obstetrician before patient discharge. Informed signed consent was obtained before completing the questionnaire (from participants aged 16 years or above, or from a parent or legal tutor in case the woman was younger than 16 years of age). To limit illiteracy bias, the obstetrician read, explained, and completed the questionnaire according to the participant’s responses. 

Differences between maternal characteristics corresponding to the three age groups were analysed using Chi-square statistical test with a significance level of *p* < 0.05. Differences in the size of Robson groups corresponding to women in middle and late adolescence were assessed using a two-sample t-test assuming unequal variances, with a significance level of *p* < 0.05. Statistical tests were not performed on the early adolescence age group because of the reduced sample size. All statistical tests were performed using Microsoft 365 data analysis option. 

## 3. Results

There were 1718 births in our department during the study period with 260 deliveries registered in the adolescent population in our institution. One hundred twenty teenagers gave birth through CS, accounting for 6.98% of all deliveries. A number of 118 participants met the inclusion criteria and formed the group of young mothers who delivered through CS (named the “study group”). 

Reported to overall adolescent deliveries from our department during the study period, CS were responsible for 47.01% of births (Table 1). For modified Robson groups 2, 5, 7, 8, and 10, all young women gave birth through CS. As regards nulliparous women (single cephalic, ≥37 weeks, in spontaneous labour), 44.59% of them delivered through CS. The same outcome was identified for 60% of the nulliparous women (singleton breech in spontaneous labour).

Further analysis of young mothers who delivered through CS showed that their mean age was 17.4 years old (min 14 years, max 19 years, std dev 1.29 years); most study participants were in their late adolescence (50%). There was a statistically significant association between maternal age and parity (chi-square test, *p* = 0.006 (Table 2)). A proportion of 5.08% of women aged 18 to 19 years old had two previous CS; relative to the entire study population, 24.57% of women had at least one previous CS, the youngest having one at 16 years of age. Comparing young mothers with a scarred uterus, we have found that there was no association between the number of previous CS (one or two) and maternal age in middle or late adolescence (chi-square test, *p* = 0.25). 

Women in the three subgroups had on average between 5 to 6.7 prenatal visits at the obstetrician (5 visits on average for the 10–14 years group, 5.78 visits for the 15–17 years group, and 6.74 visits for the 18–19 years group) based on their reported number of consultations. 

**Figure 1:**
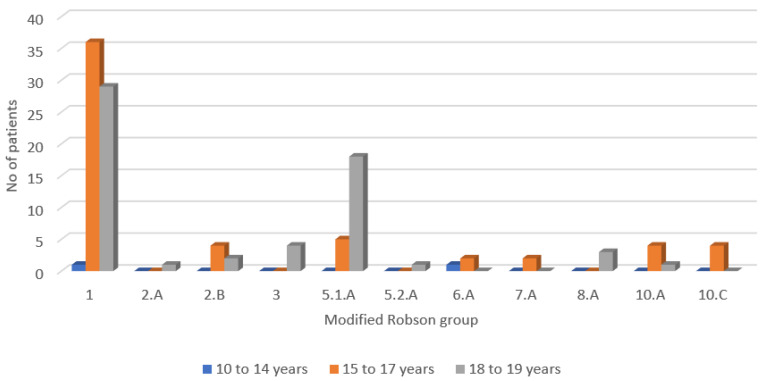
Modified TGCS distribution among age groups.

Distribution of modified Robson TGCS (Figure 1) showed the following pattern for the adolescent mothers who had CS birth: there were no women included in group 4 (multiparous, singleton, cephalic, term births) without previous CS with (4a) induced labour or (4b) prelabour CS and group 9 (transverse and oblique lies) including previous CS. The youngest participants in the report aged 14 years old had a corresponding mTGCS 1 and 6.A. Robson group 1 represented 30.50% of participants in the 15–17 years old group and 24.57% of participants in the 18–19 years old group. Group 10 was second most often identified in the middle adolescence age group, reported in 14.03% of women corresponding to that age. In the late adolescence group, 32.20% of the women were distributed in group 5. However, differences between middle and late adolescence age groups were not statistically different (*p* two tail = 0.96 using two-sample t-test assuming unequal variances).

During the study period, no participant was from group 4 or 9, irrespective of the mode of delivery. The labour induction rate was 0.84%; a proportion of 2.54% of young women had multiple gestations, and all of them delivered through CS.

There was a wide range of indications for CS among study participants. Abnormal or indeterminate foetal heart rate (FHR) tracing accounted for 28.81% of the cases; previous CS was responsible for another 24.57% of the surgical interventions. Labour dystocia (11.86%), arrest of descent (10.16%), and arrest of dilation (7.62%) were other indications observed during research. 

**Figure 2:**
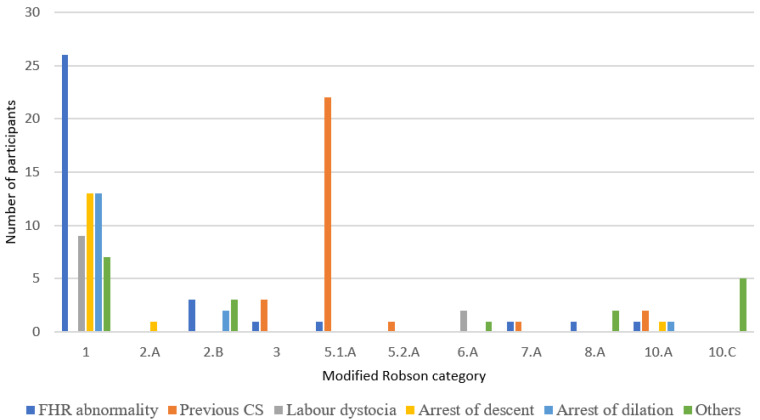
Distribution of CS indication according to modified Robson classification.

Abnormal cardiotocographic findings (38.23%) along with the arrest of descent (19.11%) and arrest of dilation (19.11%) were the most frequent indications for CS in Robson group 1 (Figure 2). Uterine scar other than post-CS was responsible for three CS deliveries (75%) in Robson group 3. Adolescents with multiple pregnancies in spontaneous labour had CS in two out of three cases due to foetal presentation dystocia. Severe preeclampsia and placental abruption were the most frequent indications for emergency CS in women with single cephalic, <=36 weeks of gestation, CS before labour (modified Robson group 10.C), determined in 20% and 40% of the cases, respectively.

Analysis of newborn parameters as presented in Table 3 reveals that the most favourable outcomes were associated with modified Robson group 5.A and 2.B: there was no admission to NICU, and mean Apgar scores were highest (10, 9.22, and 9.17), as well as mean newborn weight (3390 g, 3142.9 g, and 3165 g). Preterm births correlated with modified Robson groups 10.A and 10.C did not associate the most adverse neonatal outcomes. Neonates from mothers in modified Robson groups 8 and 7 were born at 35.3 and 35.5 weeks of gestation and required the longest admissions in NICU (11.67 days and 12.5 days on average). CS indications in both groups were related to abnormal foetal heart rate tracing; for group 7, previous CS was another indication for surgery and for group 8, breech presentation was identified in the first foetus. There was a moderate inversely proportional relation between 5-min Apgar scores and the average length of NICU admission: Pearson correlation r = −0.61. No significant correlation could be established between mean gestational age and average NICU length of stay (Pearson correlation r = −0.45).

## 4. Discussion

The assessment of CS practice represents a complex process, especially when this matter is approached at the national level. Reaching a balance between very low CS rates indicating poor overall access to life-saving facility-based maternal health interventions, and high rates suggesting inappropriate overuse of resources [12] has become a challenge for national healthcare systems. Consequently, comprehensive evaluation of each medical unit has become a feasible target, empowered by the implementation of Robson TGCS in 2001 [9].

Adolescent pregnancy and delivery have been proven to associate not only adverse pregnancy outcomes such as preterm delivery, stillbirth, maternal depression, eclampsia, and even maternal or neonatal death, but also low school achievement, increased health care costs, and living in poverty [13]. Risk factors for primary CS delivery in teenage populations have been scarcely studied but limited data is suggesting that failure to progress or descent might justify the increased risk of CS [13,14].

Some concerning aspects are rising from the results of our statistical analysis. The first one is regarding the high incidence of 44.59% of CS among teenage primiparas (≥37 weeks, in spontaneous labour), which are also responsible for 55.93% of all CS in the study population. These results highly exceed the 10–15% rates of CS considered justified by the WHO [8]. Potential contributors to the rising trends in CS are the socio-demographic factors, i.e., the dominance of rural provenience implies reduced options for school attendance and limited access to prenatal visits [4]. Unfortunately, sustained community awareness campaigns focused on adolescent sexuality, the risks of unintended pregnancies, and methods to prevent them are sporadic and lack financial support from the Romanian government [15].

TGCS does not explain why CS is performed [16] but provides a specific approach for etiological analysis. It is crucial to identify the real reasons behind this elevated proportion of CS performed in the adolescent population. The significant rise in CS rates among low-risk women of all ages over the last two decades has been confirmed to affect teenagers as well [14]. This is in accordance with our results showing that most CS in young women were Robson group 1. 

This issue represents a determinant factor for the 24.57% of 16 to 19 years old mothers who present themselves with at least one previous CS, making them absolute candidates for another delivery through CS as seen above. In our report, Robson group 5 accounted for 20.33% of all CS, making it a main contributor to overall CS rate along with group 1. Other studies have shown equivalent results [17]. The long-term consequences of repeat CS imply significant maternal and infant morbidity and mortality as a result of abnormal placentation and uterine rupture [14], thus justifying worldwide guidelines to promote individual patient assessment, with one previous CS not being necessarily an indication for another one [18]. However, medical institutions’ policies are mostly responsible of favouring prelabour CS in women with a scarred uterus.

Another point to be discussed emerges from the fact that the most unfavourable neonatal health indicators, mostly related to NICU length of stay, were not observed among Robson group 10 (premature single cephalic deliveries) but more often in specific obstetrical circumstances such as women with multiple gestations (Robson group 8) or multiparas with foetus in breech presentation (Robson group 7). The fact that most of these young women also associated premature birth is a relevant point to take into consideration since prematurity is a confounding variable in interpreting the related results. However, the CS rate in both group 7 and 8 reached 100% even if the relative contribution to overall CS was low due to small group size, while in group, 10 overall CS rate was lower than 100%. CS could be taken in consideration as a complementary responsible factor for neonatal morbidity in group 7 and 8, but previous studies did not identify significant differences in foetal morbidity in twin pregnancies with planned CS versus vaginal delivery [19]; in group 7, 50% of CS were performed on scarred uterus. Adjusting for confounding variables like obstetrical complications was not necessary since no maternal medical conditions were identified in groups 7 and 8. Overall, the newborns with higher 5-min Apgar scores were more likely to have a reduced number of admission days in the NICU (Pearson correlation r = −0.61). These results concerning NICU admission and high CS rates in group 7 and 8 are similar to recent studies [18].

Presentation of most frequent indications for CS identified in teenage mothers participating in this research reflects the consistent observations from literature, acknowledging that dystocia, foetal distress, breech presentation, and a repeat CS are the four major determinants of CS rates [20]. Additionally, while the range of indications has come to broaden considerably, we agree with other researchers who anticipated almost 40 years ago that patients and their caregivers developed a low tolerance for foetal risk associated with vaginal birth [21]. This premise is exponentially enhanced if patients are teenage women preparing to give birth. Robson group 10 added 7.61% to the overall CS rate, illustrating a high number of risk pregnancies, mainly in the middle adolescent group of women. Sanchez et al. proved that preeclampsia was associated with the occurrence of CS, owing to higher rates of CS in Robson groups 1, 5, and 10 [22]. We have identified preeclampsia and placental abruption as main indications for CS in Robson group 10, mainly in middle adolescence young mothers.

Despite previous concerns about an immature pelvic structure, investigations demonstrate that adolescents are more likely to have a successful vaginal delivery [13]. This leads to a necessity for differential intrapartum management according to maternal age. Acknowledging that slower cervical change in the first stage of labour and shorter second stage which were observed in teenage mothers [13,14] are possible obstetric scenarios should become a reality. Promoting vaginal deliveries is also cost-beneficial since a recent analysis performed in Brazil showed that natural childbirth was more cost-effective than elective CS for primiparous normal risk pregnant women. This conclusion did not apply, however, for multiparous women with previous uterine scar [23]. Compared to a previous investigation performed in 2017 in our clinic, which revealed that young women were more likely to give birth by CS at any age group [24], our present results show a modest but encouraging increase in vaginal delivery rates among teenagers. Nevertheless, labour dystocia, arrest of descent, and arrest of dilation are still concerning issues in Robson group 1, accounting for 51.47% of CS. Segregation between foetal reasons and dystocic aetiology of CS indications in spontaneous labour needs further research, as do indications for induction of labour. Although performed in 0.86% of cases, indications for induction of labour are difficult to define and lead to inconsistency in their use [25]. Grouping of indications has been proposed [25] including those for pre-labour CS, even if a degree of overlap has been anticipated. 

Romania is missing from the European map of data for the TGCS, as the Euro-peristat project, which aims to monitor perinatal health indicators across the continent, has recently been noted [3]. This research is a contribution towards our country stepping up its reporting system of obstetrical practice through the mTGCS.

The strength of this study is the preliminary focus on adolescent mothers and their mode of delivery. Also, analysing CS rate in this pregnant population using the mTGCS, and interpreting the results in connection to specific CS indications, offers a new perspective on the etiological aspects of this issue. There is a lack of funding for studies and research meant to identify the specific needs and expectations of Romanian adolescents [15]. Good quality of care should be assured according to societal dynamics; the pregnant population of patients of 2021 is not the same as the one from the year 2000, and therefore obstetric practice should adapt its skills and performance to current requirements. 

A limitation of this study is related to the limited period when mTGCS has been implemented, with this leading to a reduced statistical power (especially for women in the early adolescence group). This group of participants has been considered as part of the overall “adolescent group” but were not included in teenage subgroups-related statistical tests. Another feature we are trying to surpass is the single centre implementation of Robson criteria. Both aspects are being improved by the following interventions: continuous implementation of mTGCS in our department since 1st of January 2018 [11] and by sending proposals to other obstetrical departments in our country to engage in registering and reporting Robson TGCS. 

In the modern era of free access to information and the context of the current spread of litigious exposure, reducing CS rates can be a challenging task for medical practitioners. even if some authors strongly agree that the CS rate by itself is not a reflection of the quality of care [20].

A previous Romanian study from a different geographical region identified teenage mothers to associate lower risk for CS compared to their older counterparts, concluding that labour in <20-year-olds may be treated similarly to that in adult women [5].

However, the researchers conducting this study suggest a more cautious attitude; different intrapartum practice pattern should be mandatory in Romanian teenage pregnant women. Researchers mention the first stage of labour holds the key to adolescents’ reduced CS rates [10]. Although feasible to implement, TGCS has its challenges and difficulties [26] but practitioners can overcome this drawback. While Switzerland has already been successfully implemented automated methods to categorize patients in complex groups using routinely collected health data [27], in our department, we have managed to classify women by adding their corresponding class in the birth registry. 

TGCS is highly capable of classifying mothers and their corresponding infants into meaningful groups concerning their outcome [27]. Interpreting the results should be based on the observation that CS rates differ from tertiary hospitals to general hospitals, clinics, and province units (as seen in the Korean healthcare system) [28].

## 5. Conclusions

Our study served as a technical concept and managed to identify CS trends in young mothers giving birth in our department. The authors gained insight into adolescents at risk of repeat CS. We have identified that the need for future focus on obstetrical management is mandatory in Robson groups 7 and 8. In these two classes, adverse neonatal outcomes must be investigated after the exclusion of prematurity as a confounding factor. We also found that strategies to reduce CS rates should be submitted especially to Robson group 1, which comprised the higher proportion of CS among adolescent mothers. 

Detailed research on CS indication grouping depending on the mode of labour-onset, maternal or foetal aetiology, individualized itself as a potential source of obstetrical outcome guidance.

Implementing and reporting Robson TGCS is possible as proven above, particularly since there is much potential for digital solutions to reduce the overload of responsibilities of medical personnel.

## Figures and Tables

**Table 1:** Robson mTGCS in the adolescent population who delivered between 1 March 2020 and 1 March 2021 in “St. Pantelimon” Emergency Hospital.

mRobson Group	Description of mTGCS	No of Births *	No of CS	Relative Size of Group (%) ^1^	CS Rate: Each Group (%)	Absolute Contribution on the Overall CS Rate (%) ^2^	Relative Contribution on the Overall CS Rate (%) ^3^
1	Nulliparous women, single cephalic, ≥37 weeks, in spontaneous labour	148	66	58.96	44.59	26.29	55.93
2.A	Nulliparous women, single cephalic, ≥37 weeks, induced labour	1	1	0.40	100	0.39	0.84
2.B	Nulliparous women, single cephalic, ≥37 weeks, CS before labour	6	6	2.39	100	2.39	5.08
3	Multiparous women (excluding prev. CS), single cephalic, ≥37 weeks, in spontaneous labour	43	4	17.13	9.3	1.59	3.38
4	Multiparous, singleton, cephalic, term births without previous CS with (4a) induced labour or (4b) prelabour CS	0	0	0.00	0	0	0
5.1.A	Multiparous women, one previous CS, single cephalic, ≥37 weeks, in spontaneous labour	23	23	9.16	100	9.16	19.49
5.2.A	Multiparous women, at least two previous CS, single cephalic, ≥37 weeks, in spontaneous labour	1	1	0.40	100	0.39	0.84
6.A	All nullipara breeches in spontaneous labour	5	3	1.99	60	1.19	2.54
7.A	All multipara breeches (including previous CS) in spontaneous labour	2	2	0.80	100	0.79	1.69
8.A	All multiple pregnancies in spontaneous labour	3	3	1.20	100	1.19	2.54
9	Transverse and oblique lies, including previous CS	0	0	0.00	0	0	0
10.A	All single cephalic, <=36 weeks (including previous CS) in spontaneous labour	15	5	5.98	33.33	1.99	4.23
10.C	All single cephalic, <=36 weeks (including previous CS), CS before labour	4	4	1.59	100	1.59	3.38
Total		251	118	100	47.01	47.01	99.94

* there were 260 teenage deliveries in our Department during the study period but 9 were excluded from the analysis (incomplete data or women did not consent to participate in the study) ^1^ (Number of deliveries in the group)/(total number of deliveries); ^2^ (Number of CS in the group)/(total number of deliveries); ^3^ (Number of CS in the group)/(total number of CS).

**Table 2:** Characteristics of adolescents by age group.

	Age 15–17 Years		Age 18–19 Years		Total	Chi-Square Test
	*n*	%	*n*	%	*n*	*p* Value
No of Participants	57	48.30	59	50.00	118	
Residence						
Urban	17	29.82	20	33.89	38	0.637
Rural	40	70.12	39	66.10	80	
Years of Schooling						
0 years	3	5.26	3	5.08	6	0.023
1–8 years	43	75.43	31	52.54	76	
9–10 years	8	14.03	11	18.64	19	
10–12 years	3	5.26	14	23.72	17	
Parity						
1	48	84.21	35	59.32	85	0.006
2	9	15.78	20	33.89	29	
3	0	0.00	4	6.77	4	
Previous CS						
0	49	85.96	38	64.40	89	0.016
1	8	14.03	18	30.50	26	
2	0	0.00	3	5.08	3	
Type of Pregnancy						
single	57	100.00	56	94.91	115	0.084
multiple	0	0.00	3	5.08	3	
Fetal Presentation						
Cephalic	53	92.98	58	98.30	112	0.158
Breech	4	7.01	1	1.69	6	
Maternal Length of Stay						
<5 days	19	33.33	27	45.76	46	0.171
≥5 days	38	66.66	32	54.23	72	

**Table 3:** Statistical description of newborn outcomes in modified Robson groups.

Modified Robson Group	Gestational Age (Weeks)			Newborn Birth Weight (g)			Apgar Score			NICU Length of Stay (Days)		
	Min	Max	Mean	Min	Max	Mean	<7	≥7	Mean	Min	Max	Mean
1	37	41	38.97	2110	4750	3145.91	1	65	9.14	0	11	0.76
2.A	38	38	38	3310	3310	3170	0	1	9	0	0	0
2.B	38	42	39.5	2700	3770	3165	0	6	9.17	0	0	0
3	37	39	37.75	2020	3560	3050	0	4	8.5	0	15	3.75
5.1.A	37	41	38.08	2470	3980	3142.92	0	23	9.22	0	0	0
5.2.A	38	38	38	3390	3390	3390	0	1	10	0	0	0
6.A	37	38	32.67	2930	3320	2216.67	0	3	8	0	0	0
7.A	27	34	35.5	1200	2520	2615	0	2	8.5	10	15	12.5
8.A	33	36	35.33	1850	2710	2200	0	3	8.33	0	24	11.67
10.A	33	36	34	1930	2870	2412	0	5	8.2	0	40	10
10.C	32	36	36.75	1750	2860	2530	1	3	8.33	0	37	9.25

## Data Availability

Study database is available on request.
